# *Borrelia recurrentis* in Head Lice, Ethiopia

**DOI:** 10.3201/eid1905.121480

**Published:** 2013-05

**Authors:** Amina Boutellis, Oleg Mediannikov, Kassahun Desalegn Bilcha, Jemal Ali, Dayana Campelo, Stephen C. Barker, Didier Raoult

**Affiliations:** Marseille UniversitA(c), Marseille, France (A. Boutellis, O. Mediannikov, D. Raoult);; Campus Commune UniversitA(c) Cheikh Anta Diop-Institut de Recherche pour le DA(c)veloppment d'Hann, Dakar, SA(c)nA(c)gal (A. Boutellis, O. Mediannikov, D. Raoult);; University of Gondar, Gondar, Ethiopia (K.D. Bilcha, J. Ali);; University of Queensland, Brisbane, Queensland, Australia (D. Campelo, S.C. Barker)

**Keywords:** *Borrelia recurrentis*, *Bartonella quintana*, relapsing fever, trench fever, head lice, body lice, Ethiopia, bacteria, Rickettsia

## Abstract

Since the 1800s, the only known vector of *Borrelia recurrentis* has been the body louse. In 2011, we found *B. recurrentis* DNA in 23% of head lice from patients with louse-borne relapsing fever in Ethiopia. Whether head lice can transmit these bacteria from one person to another remains to be determined.

Humans are the sole hosts of the pubic louse (*Pthirus pubis*), the body louse (*Pediculus humanus humanus*), and the head louse (*Pediculus humanus capitis*) (*1*). The body louse can transmit the following life-threatening forms of bacteria to humans: *Rickettsia prowazekii*, which causes epidemic typhus; *Bartonella quintana*, which causes trench fever; and *Borrelia recurrentis*, which causes louse-borne relapsing fever (*2*). Recently, DNA from *B. quintana* has been found in head lice from Nepal (*3*), the United States (*4*), France (*5*), Senegal (*6*), and the highlands of Ethiopia (Gibarku and Tikemit Eshet) (*7*). Louse-borne relapsing fever is among the top 10 causes of hospital admissions in Ethiopia and is associated with substantial illness and death (*8*). Infection of head lice with *B. recurrentis* or *R. prowazekii* has not been reported. Our aim was to assess the presence of *Borrelia, Rickettsia,* and *Bartonella* spp. in head lice and body lice from persons in the highlands of Ethiopia, where an outbreak of relapsing fever is ongoing.

## The Study

In August 2011, we enrolled 24 patients (23 male, 1 female) at Bahir Dar Hospital, Ethiopia, whose blood smears were positive for *Borrelia* spp. by microscopy with Giemsa staining. After receiving permission from the patients, we collected samples of head and body lice by searching their heads and clothing. Lice were randomly selected, preserved in 100% ethanol, and taken to the reference center at Marseille UniversitA(c), Marseille, France. Each louse was rinsed 2A- in sterile water. Genomic DNA was extracted by using the QIAamp DNA FFPE Tissue Kit (QIAGEN, Hilden, Germany), as recommended by the manufacturer, and stored at �^'20A�C.

Quantitative real-time PCR (qPCR) was performed by using primers and probes that targeted a portion of the *Bartonella* 16S�?"23S intergenic transcribed spacer region and a specific *B. quintana* gene, *yopP*, which encodes for a putative intracellular effector (*5*). For a specific *R. prowazekii* gene, we used previously described primers and probes (*9*), and for *Borrelia* spp., we used previously described primers and probes selective for the 16S rRNA gene (*10*). To confirm the positive qPCR results, we performed a standard PCR that used primers for an intergenic spacer region between the 16S rRNA gene and a gene encoding a hypothetical protein of *B. recurrentis*. The primers used for this experiment were B.rec F: 5�?�-TTCGCCACTGAATGTATTGC-3�?� and B.rec R: 5�?�-TGCCAATGTTCTTGTTGGTC-3�?� (*11*). Uninfected body lice (Orlando strain) were used as negative controls for each test.

Among the 24 patients, 11 had head and body lice, 11 had body lice only, and 2 had head lice only. Classification of lice was based on phenotype (head lice, black; body lice, gray), ecotype (found on head and hair or in clothing), and cytochrome b genotype (clade) (data not shown) (*1*). Some head lice were found on the patients�?(tm) clothing, specifically on collars and hats.

Totals of 35 head lice and 62 body lice were tested individually. *Borrelia* spp. DNA was found in 8 (23%) head lice from 5 patients and in 25 (40%) body lice from 15 patients (Table). *B. quintana* DNA was found in 1 (3%) head louse and in 7 (11%) body lice. *Borrelia* spp. and *B. quintana* were found in 5 (8%) body lice from 3 patients (Table). DNA from *R. prowazekii* was not found in any of the 97 lice.

**Table:** Results of quantitative real-time PCR analysis of head lice and body lice from 24 patients, Bahir Dar, Ethiopia, 2011*

Patient no.	Patient sex	No. head lice	No. body lice	*Bartonella quintana*			*Borrelia recurrentis*	
In head lice	In body lice	In head lice	In body lice
Head and body lice								
1607007	M	3	2	0	0		0	0
1607008	M	2	1	0	0		1 (C_t_ 34)	1 (C_t_ 25)
1607009	M	1	3	0	0		0	0
16070010	M	3	3	0	1 (C_t_ 35)		0	1 (31)
1807002	M	2	3	0	0		2 (C_t_ 34)	3 (C_t_ 35)
1907002	M	3	3	0	0		1 (C_t_ 33)	3 (C_t_ 30)
2007001	M	3	2	1 (C_t_ 32)	0		0	1 (C_t_ 35)
2007002	M	3	3	0	0		0	2 (C_t_ 34)
2107001	M	3	3	0	0		0	2 (Ct 27)
2107002	M	3	3	0	0		0	1 (C_t_ 34)
2307002	M	3	3	0	3 (C_t_ 27)		2 (C_t_ 34)	2 (C_t_ 31)
Total	11	29	29	1 (3.44%)	4 (13.8%)		6 (20.70%)	16 (55.17%)
Body lice only								
1607001	M	0	3	0	0		0	1 (C_t_ 29)
1607002	M	0	3	0	0		0	1 (C_t_ 35)
1607003	M	0	3	0	0		0	1 (C_t_ 21)
1607004	M	0	3	0	2 (C_t_ 36)		0	2 (C_t_ 27)
1607005	M	0	3	0	0		0	0
1607006	M	0	3	0	0		0	0
1707001	M	0	3	0	0		0	1 (C_t_ 35)
1907003	M	0	3	0	0		0	3 (C_t_ 31)
2407001	F	0	3	0	0		0	0
2407003	M	0	3	0	0		0	0
2407004	M	0	3	0	1 (C_t_ 36)		0	0
Total	11	0	33	0	3 (9.0%)		0	9 (27.27%)
Head lice only								
1807001	M	3	0	0	0		2 (C_t_ 31)	0
1907001	M	3	0	0	0		0	0
Total	2	6	0	0	0		2 (33.33%)	0

*C_t_, cycle threshold.

For the 11 patients who were infested with head and body lice (29 of each type), prevalence of *Borrelia* spp. DNA was significantly higher among the body lice than among the head lice (16 [55%] of 29 vs. 6 [21%] of 29; p = 0.006) (Table). *B. quintana* DNA was found in 1 (3%) head louse and in 4 (14%) body lice, but the difference was not significant. For the 11 patients infested with body lice only, 9 (27%) of 33 body lice were positive for *Borrelia* spp. and 3 (9%) were positive for *B. quintana*. For the 2 patients infested with head lice alone, 2 (33%) of 6 head lice were positive for *Borrelia* DNA. The *Borrelia* DNA that was in 4 head lice and 5 body lice (collected from 9 patients) was then used to identify the species of *Borrelia* that infected these patients. For this identification, we performed a pairwise comparison of the intergenic spacer sequence from the putative *Borrelia* species in these patients with *B. recurrentis, B. duttonii*, and *B. crocidurae* sequences from GenBank; results showed 100%, 97%, and 93% similarities, respectively (GenBank accession nos. JX126797�?"JX126805).

We detected *B. recurrentis* DNA in head and body lice by using qPCR and confirmed these results by sequencing the amplicons. Among patients infested with head and body lice, the numbers of body lice infected with *B. recurrentis* were substantially higher than the numbers of head lice infected with *B. recurrentis*; however, *B. recurrentis* DNA was also found in lice from patients infested with head lice alone. 

## Conclusions

Human head and body lice are generally thought to colonize their hosts in different ways. However, head and body lice are often both found on heavily infested persons and might migrate from head to body and vice versa (*12*). Head lice in Ethiopia are black and belong to the cytochrome b clade (genotype) C, whereas body lice are gray and belong to clade A (*7*). We also found that head lice from heavily infested patients were in physical proximity (i.e., on the collars of clothing) with body lice.

Body lice are the principal vectors of *B. recurrentis* (*2*). However, head lice can also be present in large numbers on persons with body lice because the conditions that lead to prevalent and prolonged infestations with body lice�?"such as poverty, inability to change clothes, and crowding�?"also favor head lice (*5*). We hypothesize that in patients who are simultaneously infested with both types of lice, the head lice might be contaminated with blood containing *Borrelia* spp.

The transmission of relapsing fever to humans occurs by the rupturing of a louse and subsequent inoculation by scratching because *Borrelia* spp. are found in the hemolymph of the insect. Recently, however, viable *B. recurrentis* was found in lice excrement, which implies that the organism might return to the gut from the hemolymph (*13*).

In conclusion, head lice from patients with louse-borne relapsing fever were infected with *B. recurrentis* and *B. quintana.* Whether head lice can transmit these pathogenic bacteria from person to person remains to be explored. To determine whether *B. recurrentis* and *B. quintana* occur in head lice, epidemiologic studies of head lice collected from more patients with louse-borne relapsing fever and trench fever should be conducted.
